# Supplementary material for: Effects of the Developing and Using a Model to Predict Dengue Risk Villages Based on Subdistrict Administrative Organization in Southern Thailand
Source: Int J Environ Res Public Health. 2022 Sep 22;19(19):11989. doi: 10.3390/ijerph191911989 (PMC9565314; doi:10.3390/ijerph191911989)
Supplement: Supplementary file 1 [file ijerph-19-11989-s001.zip › ijerph-1891863-supplementary.pdf]

Keawsan dengue model: The Computer program for risk dengue village prediction
(https://Nakhonsi.denguelim.com)

The computer programme for supporting village dengue risk prediction was developed based on appropriate community context. The programme uses web application relating disease severity factors in the past five years and outbreak opportunity factors. The programme modules include dengue prevention responsibility in PCUs for district public health officials and administrators. The programme is easy to use by public health officers and all stakeholders in the community. The advantages of the programme include the ability to record, collect, and easily report data to predict village dengue outbreak risk quickly and the potential for several dimensions of presentation at village, PCU, and district levels.

1. Preparing risk dengue village, all stakeholders (Public health providers of PCUs of Sub-district) were meeting for understanding and preparing data for risk prediction.

1.1 They understood regarding with the risk dengue village prediction criteria (RDVPC) of *Keawsan* Model

The prediction criteria for village dengue risk involved two aspects consisting of six factors; disease severity aspect (three factors) and outbreak

opportunity aspect (three factors) which integrated epidemiology, entomology, and community activities.

1. Dengue severity aspect (DSA)

1.1 Endemic village factor (EVF) was based on the village dengue incidence rate in the previous 5 years. This criterion showed several factors of dengue incidence. The weight of the value assigned to an endemic village showed that the more the number of years of dengue incidence reporting, the higher the points. For example, a village with a weight value of 3 points had dengue incidence in 3 out of 5 years.

1.2 Dengue herd immunity factor (DHIF) refers to the average morbidity rate in the previous 5 years. According to the natural course of dengue infection, the immune system is the body's primary defence against the virus. When someone is infected with dengue, the innate and adaptive immune responses together fight the virus. The B cells produce antibodies that specifically recognise and neutralise the foreign viral particles, and cytotoxic T cells recognise and kill infected cells with dengue virus. People who are infected subsequently with a different dengue virus type may experience "antibody-dependent enhancement," a condition that occurs when the immune response worsens dengue clinical symptoms, increasing the risk of severe dengue [1]. Antibody-dependent enhancement of dengue infection assumes a level of immunity in the community showing that the higher the outbreaks in the area, the higher is the herd immunity, compared to low level outbreak areas. Therefore, areas with high dengue risk in the past 5 years should

have low outbreak in the following year [2]. For example, a weight value point in risk in a village corresponds to an average morbidity rate in 5 years of more than 200/100,000 populations.

1.3 Current morbidity rate factor (CMRF) indicates the past 5-year median rate. According to the dengue outbreak based on dengue virus serotypes and the immunology in the risk area, with a low dengue incidence rate, in the following year, the area is at high-risk of an outbreak. When the current morbidity rate is less than the past 5-year median morbidity rate, the area is at high-risk of dengue outbreak. For example, the weighting risk factor value is five points in a village with a current morbidity rate less than the past 5-year median morbidity rate (-50%).

2. Dengue outbreak opportunity aspect (DOOA)

2.1 Population movement factor (PMF) includes factors related to tourists and/or industrial areas where movement can enhance dengue virus transmission. Movement patterns of people and spatial heterogeneity of human activities influence dengue outbreak.[3] A study devised three levels of risk values for villages.[2] Then, this study, gave score 3 point for a village which had “high population movements in a village with camp workers, factories, students studying outside the area, petrol stations, shops, prisons, parks, large schools, and markets”, 2 point for moderate movement of people in the village such tourist place, and a point for the village that low movement population.

2.2 Population density in village factor (PDVF) indicates the number of people per square kilometre. This model of human population density predicted dengue

outbreak[4, 5] because dengue virus transmission occurs between humans and mosquitoes. High population density is associated with increasing dengue incidence.[6, 7] A study on dengue risk assessment criteria used the population density at five levels.[2] A village with "very high population density (> 16,400 cases/ km^2)" or "very low population density (< 5,601 cases/km^2)" received five and one point, respectively, of weight value of risk.

2.3 Strengthening villages for dengue prevention activities (SVDPA) includes activities, project, or interventions for dengue prevention in participating villages. Intervention trials should measure the impact on dengue risk.[8] Five main activities related to village strengthening include:

2.3.1) Larval indices surveillance system (LISS), which collecting, analysing, interpreting, feedback, and use of data for dengue prevention activities in a village, to show the pattern of the larval indices from households to districts.[9] For a completed system, partially conducted, or unclear and no appearance; the scores were 0, 1, and 2 points, respectively.

2.3.2) Garbage management in households and communities because most water containers with larval infestation are found outside, in household garbage. Particularly, porcelain and plastic wastes are more conducive for *Ae. aegypti* reproduction.[10] Such garbage management system should reuse, recycle, and reduce. For a completed project, partially conducted, or unclear and no appearance; the scores were 0, 1, and 2 points, respectively.

2.3.3) Larval indices lower than the standard level (BI<50, HI<10, and CI<1). For village larval indices value of three points lower than the standard level, dengue outbreak depends less on mosquito density than on environmental temperature.(3) Entomological indices are used to measure dengue vector infestation in and around structures (such as, homes and buildings). However, these indices are seldom sensitive to precisely estimate dengue transmission risk or predict impending outbreaks.[2, 11, 12] The values were lower than the standard mean of the larval indices levels in March and August as reported by the Thai MoPH (BI<50, HI<10, CI<1). We estimated the larval indices at three levels as follows: "three larval indices lower than the standard value = 0 points," "one or two of three larval indices lower than the standard value = 1 point," and "three larval indices higher than the standard values = 2 points."

2.3.4) Community capacities activities refer to the village member activities for increasing the capacity for sustainable dengue prevention, at least one project per village. In an intervention study for dengue prevention [13]. There were three values for the activities for enhancing community capacity for disease prevention. For a completed project, partially conducted, or unclear and no appearance; the scores were 0, 1, and 2 points, respectively.

2.3.5) School-based dengue prevention activities refer to dengue prevention activities taking place in schools, conducted by teachers, students, and stakeholders. Schoolchildren have high incidence rate of dengue infection as a "sentinel population," [5] and the school is a high-risk area. The larval indices criteria for

schools, CI = 0. For schools in a village where children learn either a full dengue prevention project, partially conducted, or unclear and none had the scores of 0, 1, and 2 points, respectively.

The total weight value of community participation according to the five main community participation activities to assess the village dengue risk was 10 points).

The RDVPC consisted of DSA (three factors), and DOOA (three factors) are also shown in Table S1.

Table S1 Risk dengue village prediction criteria (RDVPC) consisted of 2 aspects and 6 factors

RDVPC	Point
**1. Dengue severity aspect (DSA)**	
**1.1 Endemic village factor (EVF)**	
1.1.1 Have dengue case in village area 1 year in 5 years	1
1.1.2 Have dengue case in village area 2 years in 5 years	2
1.1.3 Have dengue case in village area 3 years in 5 years	3
1.1.4 Have dengue case in village area 4 years in 5 years	4
1.1.5 Have dengue case in village area 5 years in 5 years	5
**1.2 Dengue herd immunity factor (DHIF)**	
1.2.1 Average of morbidity rate in previous 5 years more than 200/100,000 population	1
1.2.2 Average of morbidity rate in previous 5 years between 150–200/100,000	2

RDVPC	Point
population	
1.2.3 Average of morbidity rate in previous 5 years between 100–150/100,000 population	3
1.2.4 Average of morbidity rate in previous 5 years between 50–100/100,000 population	4
1.2.5 Average of morbidity rate in previous 5 years less than 50/100,000 population	5
**1.3 Current morbidity rate factor (CMRF)**	
1.3.1 Current morbidity rate less than the median morbidity rate in the past 5 years (-50%)	5
1.3.2 Current morbidity rate less than the median morbidity rate number in the 5 years (-10% to -50%)	4
1.3.3 Current morbidity rate equals the median morbidity rate in the past 5 years (+9.9% to -10%)	3
1.3.4 Current morbidity rate higher than the median morbidity rate in the past 5 years (+10% to 50%)	2
1.3.5 Current morbidity rate higher than the median morbidity rate in five	1

RDVPC	Point
years	
(+50%)	
**2. Dengue outbreak opportunity aspect (DOOA)**	
**2.1 Population movement factor (PMF)**	
2.1.1 High movement of population means people moving in the village including camp workers, factory, students studying outside the area, petrol station, shop, prison, park, large school, and market	3
2.1.2 Moderate movement of people means the village was a tourist place	2
2.1.3 Low movement of population means no movement to other areas	1
**2.2 Population density in village (PDV)**	
2.2.1 Very high population density (> 16,400 cases/square kilometre)	5
2.2.2 High population density (12,801–16,400 cases/square kilometre)	4
2.2.3 Moderate population density (9,201–12,800 cases/square kilometre)	3
2.2.4 Low population density (5,601–9,200 cases/square kilometres)	2
2.2.5 Very low population density (< 5,601 cases/square kilometres)	1
**2.3 Strengthening village for dengue prevention activities (SVDPAs)***	
2.3.1 Larval indices surveillance system (LISS)	2
2.3.2 Garbage management	2
2.3.3 Larval indices level of village	2

RDVPC	Point
2.3.4 Community capacity activities	2
2.3.5 School-based dengue prevention activities	2
**Total full score**	**33**

* Weight value of risk (point) if village no activity gave score 2 points, Partial activity gave 1 point, and completely activity gave 0 point

1.2 Preparing data following Risk dengue village prediction criteria (RDVPC)

Public health providers of each PCU prepared data two sections: (1) Data regarding the district context for programmer writing a local program for each district such as village's name, sub-district's name, PCUs' name, and public health provider's name who responsive dengue prevention of each PCU, (2) Data of dengue problem following the RDVPC such as (1) The average morbidity rate in the previous 5 years, (2) The village dengue incidence rate in the previous 5 years, (3) Morbidity rate in current year, (4) Population movement, (5) The number of people per square kilometre, (6) Village's activities, project, or interventions for dengue prevention such as larval indices surveillance system (LISS), garbage management, larval indices level of village, community capacity activities, and school-based dengue prevention activities.

2. Prediction risk dengue villages

The village-level evaluation of risk dengue areas under the responsibility of representative of all stakeholders were evaluated based on community participatory. These stakeholders were consisting from village, PCU, public health officer, schools, child development centers, temple, VHV, and SAO. They set the meeting for discuss and evaluated the level of these criteria. In case a village had two PCUs, the evaluation was conducted according to the specified criteria established during a conference wherein official representatives were tasked with solving dengue-related problems on behalf of each PCU. Furthermore, information about illness rate, fatality rate, and total population of each district was comprehensively researched.

Villages with high- and low-risk dengue were assessed using half the total scores (33 points divided from DSA 15 points and DOOA 18 points). Thereafter, the risk cut-off value was 17 points. Villages were considered high-risk if they scored 17 or more points and low-risk if they scored less than 17 points. Example, the 10 villages under the two PCUs in the *Keawsan* SAO were stratified into five HRDVs (50 %) and five LDRVs (50 %) (Table 2).

Report risk dengue village prediction
https://Nakhonsi.denguelim.com

The https://Nakhonsi.denguelim.com programme requires the following: authentication and confirmation of the real user, using username and password; and import data (input process): the risk assessment criteria for prediction. The computer program was processed to determine the risk level and report the descriptive

statistics of a village risk level percentage in each PCU risk level and compare between PCUs in the district.

System architecture diagrams

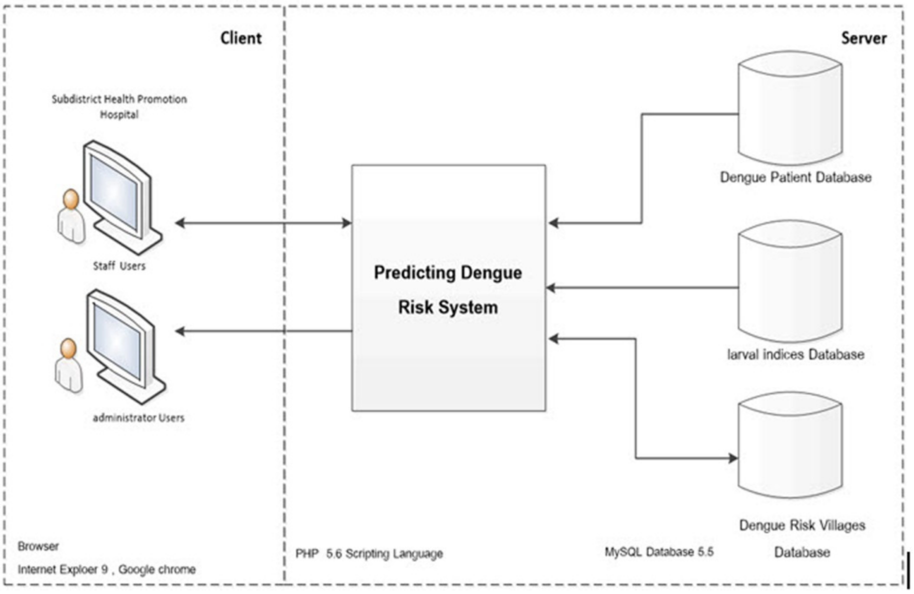

A: Input data into the program based on individual username and password

1. Open the program https://Nakhonsi.denguelim.com

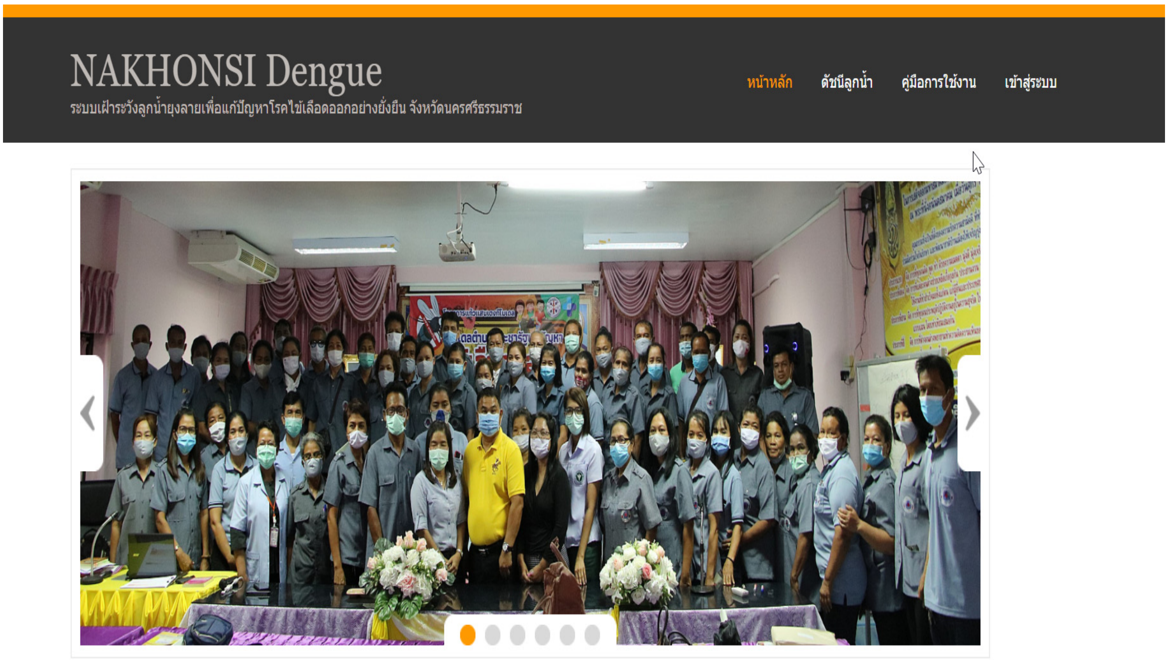

2. Sign in the program https://Nakhonsi.denguelim.com via username and Password of PCU

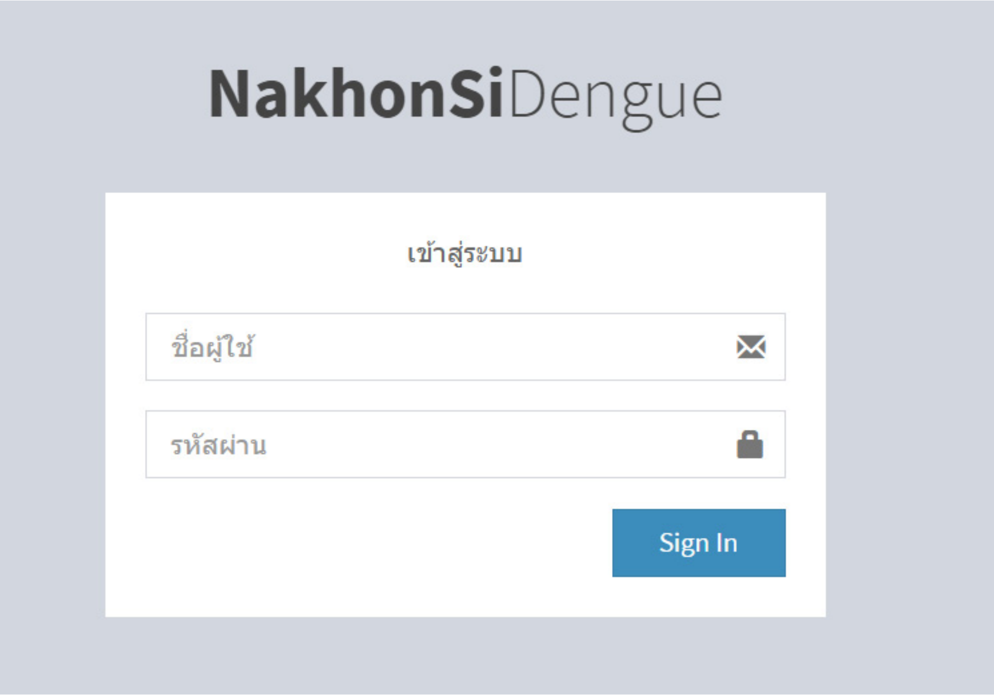

3. Set the year for prediction risk dengue village, if they predict risk dengue in 2020, the previous 5 year were 2014-2018, and current year was 2019.

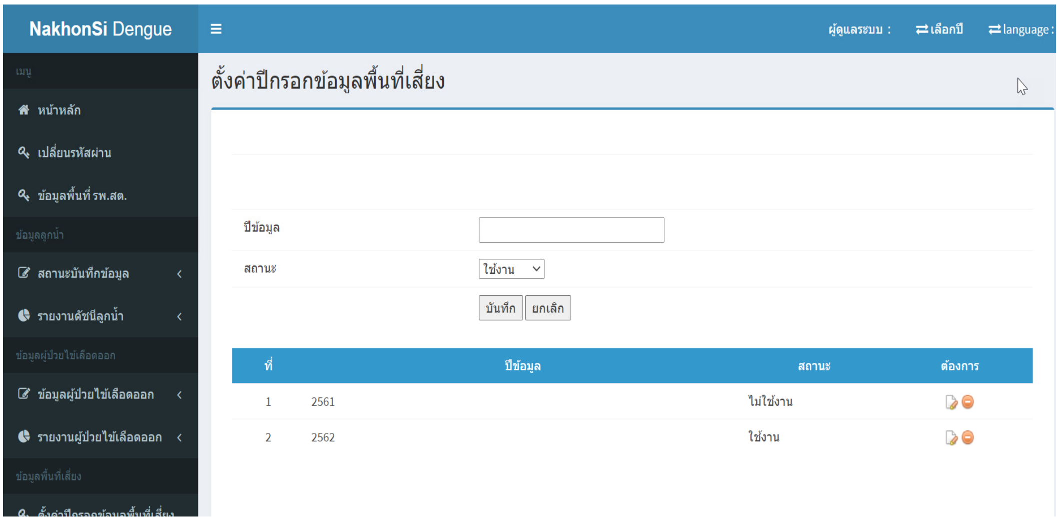

4. Entry data of average population of the past 5 years

4.1 They recorded average population in each village.

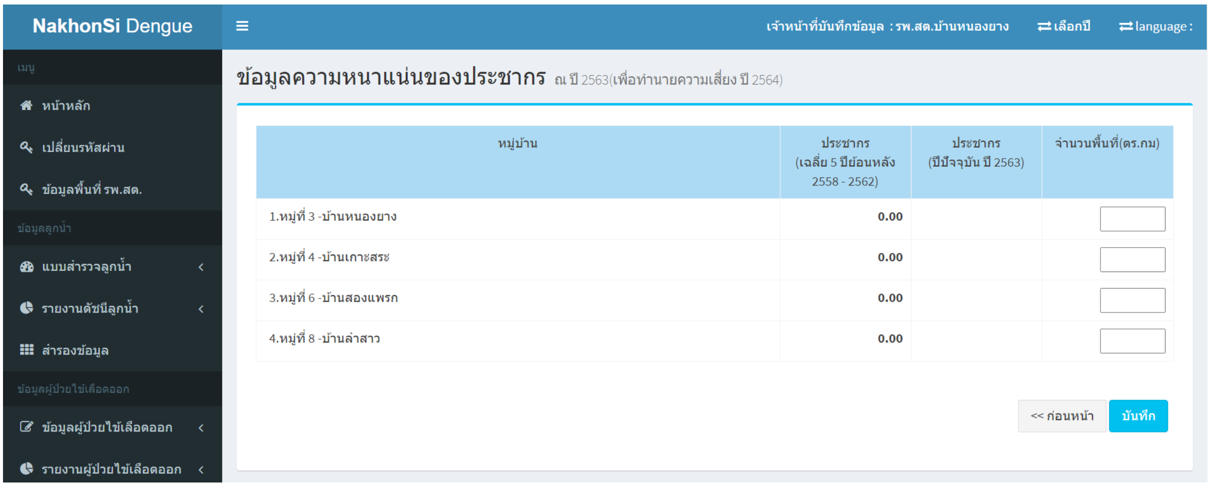

After finished click “Recorded” then, the program showed finished.

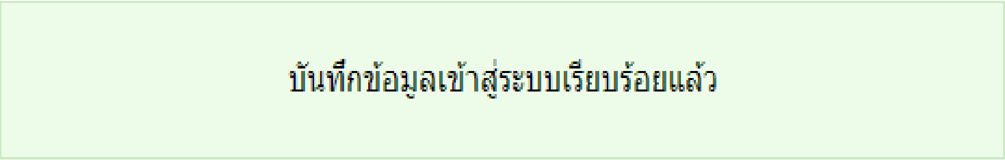

4.2 They recorded the village dengue incidence rate in the previous 5 years, and current year morbidity.

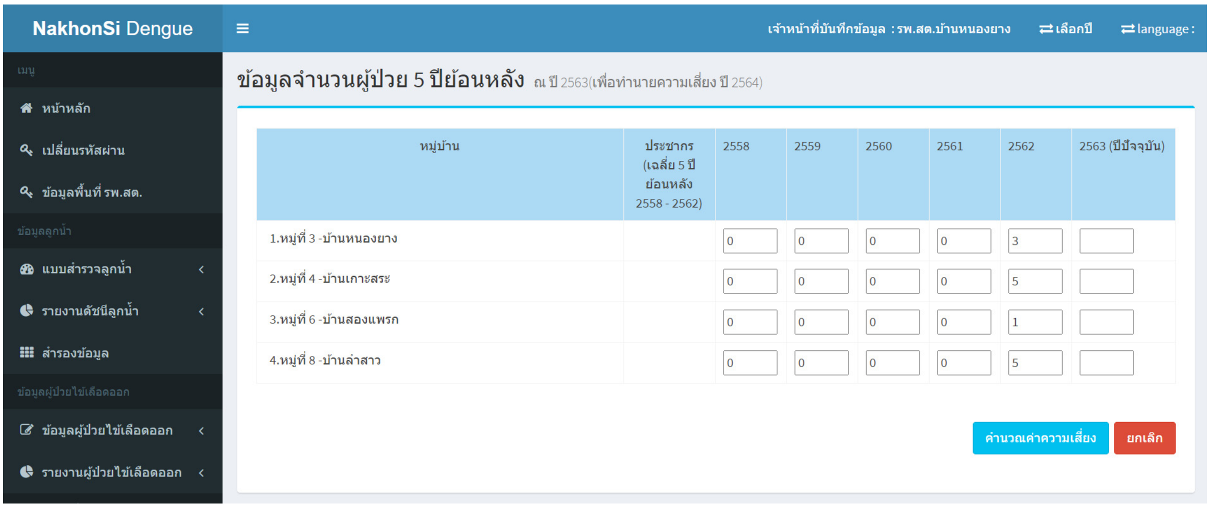

After finished click “Recorded” then, the program showed finished.

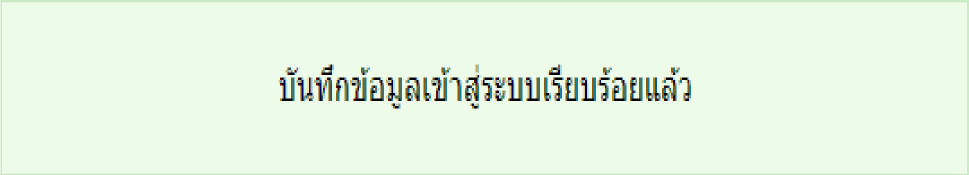

After filling in the information successfully, click the button "calculate the risk". The system will use the information to calculate the risk value in the three severity factors of dengue severity aspect:

(1) Endemic village refers to an area where an outbreak has occurred frequently in the past 5 years.

(2) Herd Immunity level was mean average of mobidity rate in the village

(3) Incidence of disease in the current year (Incidence) refers to the morbidity rate incurrent year comparing the median of morbidity.

4.3 They recorded population movement, *Population density in village factor (PDVF)* indicates the number of people per square kilometre. They recorded the score rating point following RDVPC.

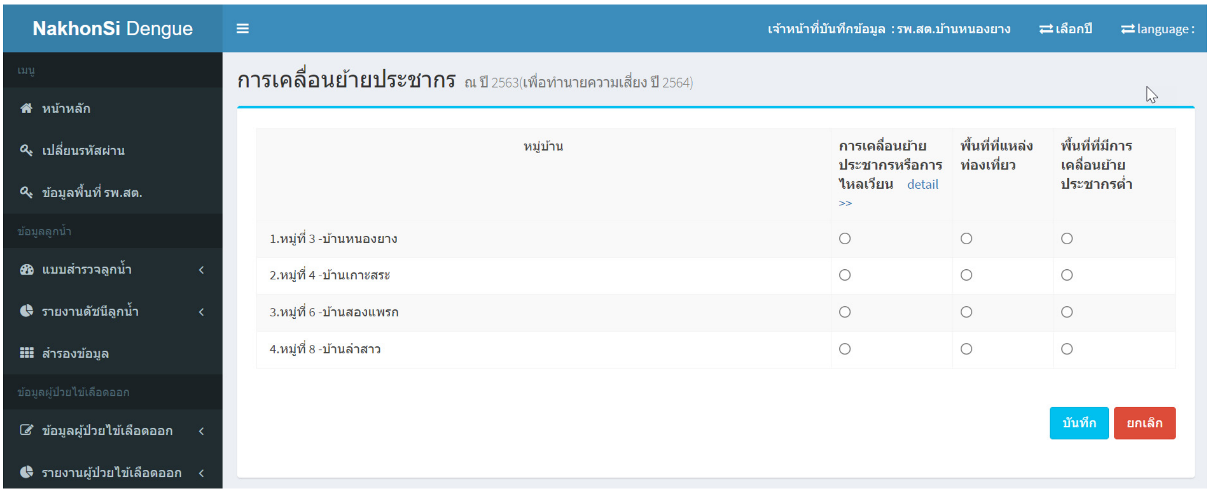

After finished click "Recorded" then, the program showed finished.

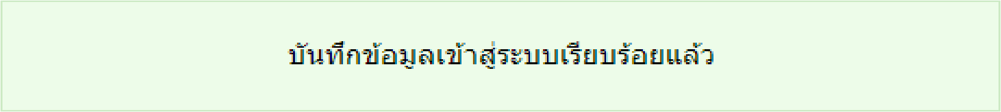

4.4 They recorded data of village's activities, project, or interventions for dengue prevention such as larval indices surveillance system (LISS), garbage management, larval indices level of village, community capacity activities, and school-based dengue prevention activities.

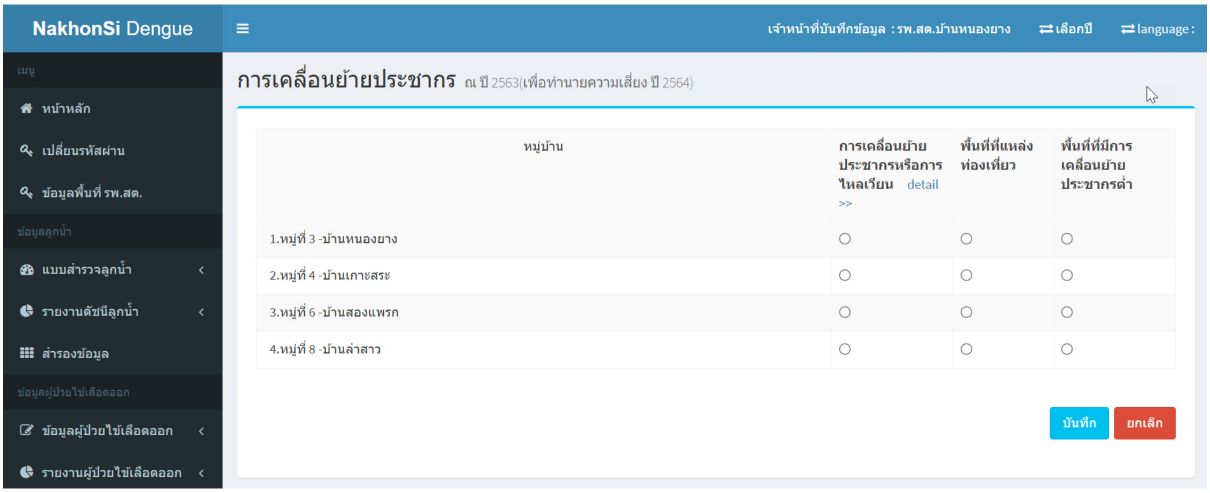

After finished click "Recorded" then, the program showed finished.

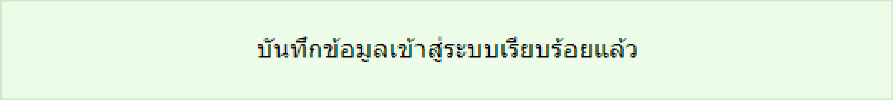

B: Report the prediction of HRDV LRDV of *Keawsan* Dengue Model

Manu of risk village are three sections such as 1) HRDV and LRDV in sub-district report of HRDV and LRDV of PCU, 2) predicting dengue risk report of each village in PCU1 3) predicting dengue risk report of each village in PCU2.

1. Example a report of HRDV and LRDV between PCU1 and PCU2 at June 2020

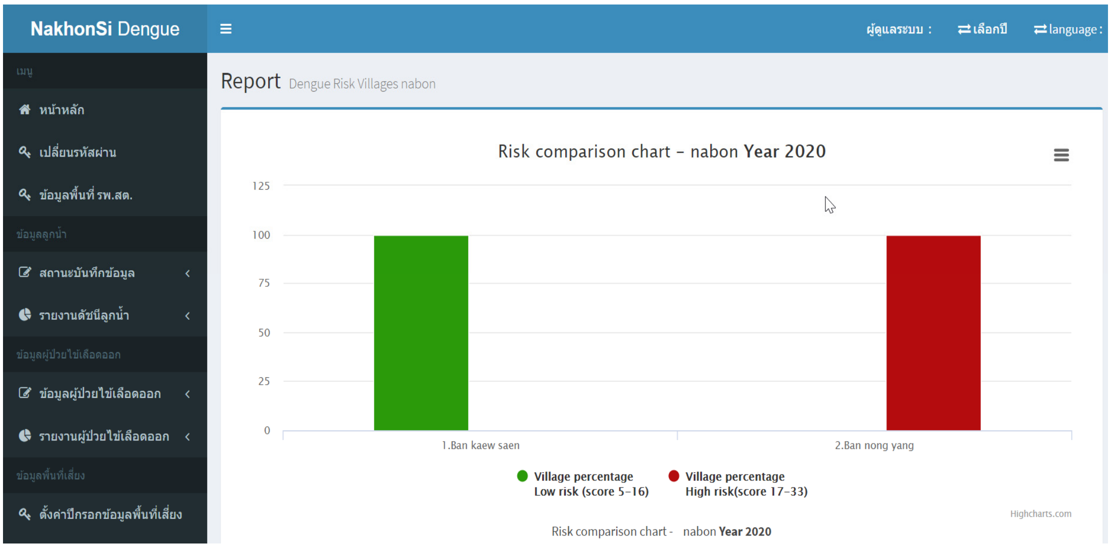

2. Example a report of predicting dengue risk village in PCU1

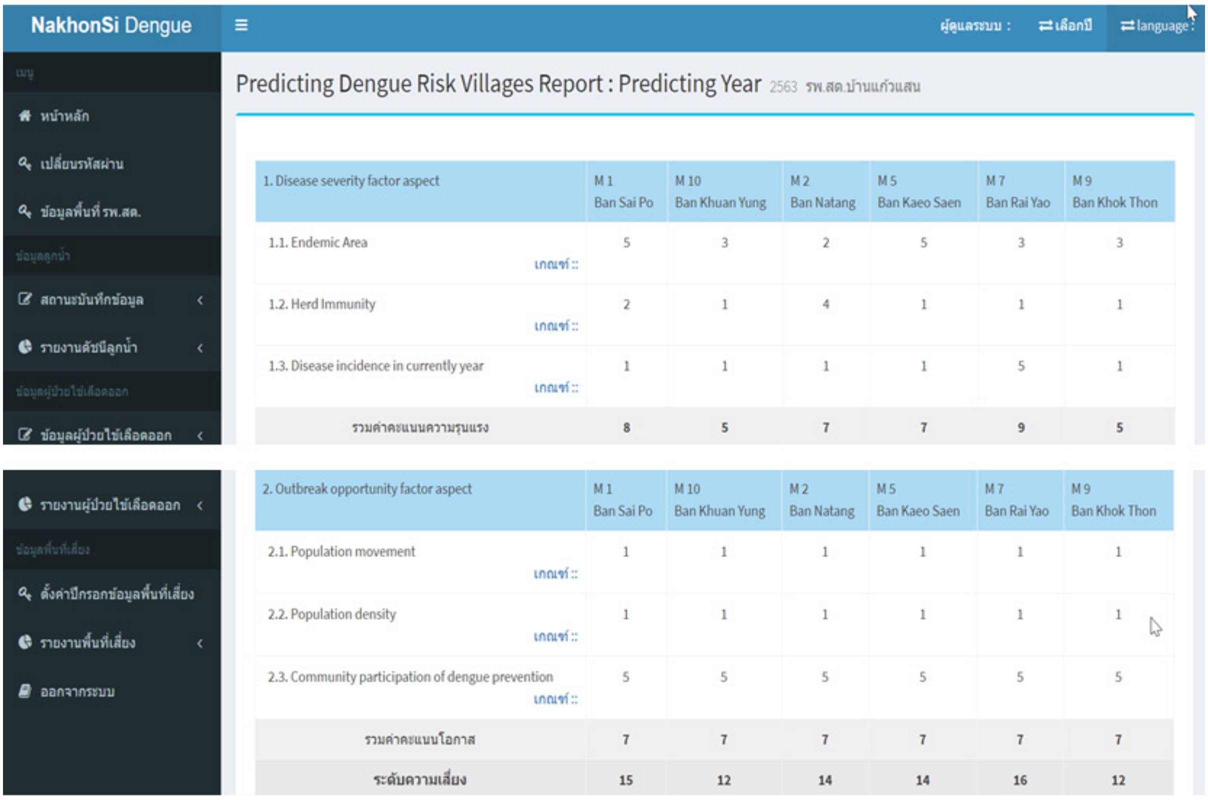

1. Disease severity factor aspect	M 1 Ban Sai Po	M 10 Ban Khuan Yung	M 2 Ban Natang	M 5 Ban Kaeo Saen	M 7 Ban Rai Yao	M 9 Ban Khok Thon
1.1. Endemic Area เกณฑ์ ::	5	3	2	5	3	3
1.2. Herd Immunity เกณฑ์ ::	2	1	4	1	1	1
1.3. Disease incidence in currently year เกณฑ์ ::	1	1	1	1	5	1
รวมค่าคะแนนความรุนแรง	8	5	7	7	9	5

2. Outbreak opportunity factor aspect	M 1 Ban Sai Po	M 10 Ban Khuan Yung	M 2 Ban Natang	M 5 Ban Kaeo Saen	M 7 Ban Rai Yao	M 9 Ban Khok Thon
2.1. Population movement เกณฑ์ ::	1	1	1	1	1	1
2.2. Population density เกณฑ์ ::	1	1	1	1	1	1
2.3. Community participation of dengue prevention เกณฑ์ ::	5	5	5	5	5	5
รวมค่าคะแนนโอกาส	7	7	7	7	7	7
ระดับความเสี่ยง	15	12	14	14	16	12

3. Example a report of predicting dengue risk village in PCU2

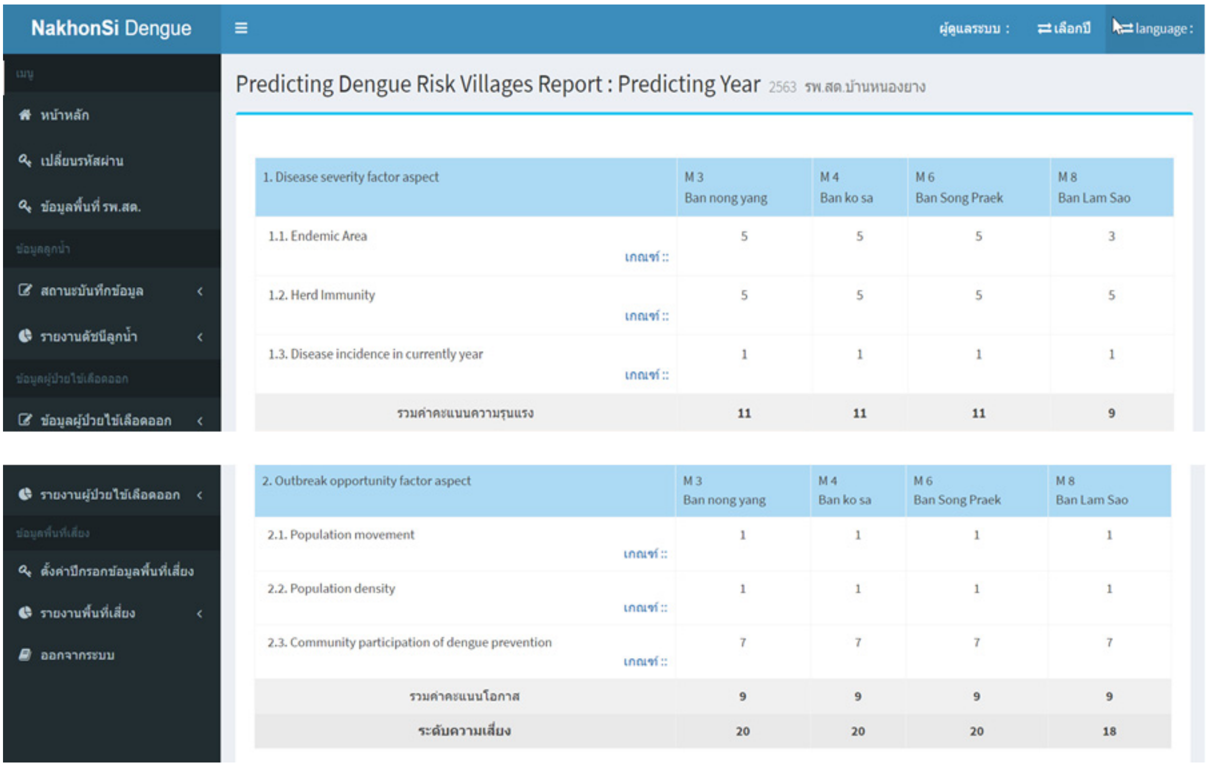

Predicting Dengue Risk Villages Report : Predicting Year 2563 รพ.สต.บ้านหนองยาง

1. Disease severity factor aspect	M 3 Ban nong yang	M 4 Ban ko sa	M 6 Ban Song Praek	M 8 Ban Lam Sao
1.1. Endemic Area เกณฑ์ ::	5	5	5	3
1.2. Herd Immunity เกณฑ์ ::	5	5	5	5
1.3. Disease incidence in currently year เกณฑ์ ::	1	1	1	1
รวมค่าคะแนนความรุนแรง	11	11	11	9

2. Outbreak opportunity factor aspect	M 3 Ban nong yang	M 4 Ban ko sa	M 6 Ban Song Praek	M 8 Ban Lam Sao
2.1. Population movement เกณฑ์ ::	1	1	1	1
2.2. Population density เกณฑ์ ::	1	1	1	1
2.3. Community participation of dengue prevention เกณฑ์ ::	7	7	7	7
รวมค่าคะแนนโอกาส	9	9	9	9
ระดับความเสี่ยง	20	20	20	18

References

1. Tang B, Xiao Y, Sander B, Kulkarni MA, RADAM-LAC Research Team, Wu J. Modelling the impact of antibody-dependent enhancement on disease severity of Zika virus and dengue virus sequential and co-infection. R Soc Open Sci 2020;7.
2. Bureau of Knowledge Management DoDC, Thai Ministry of Public Health,. Manual of assessment for center of disease control: District Health System and Disease Control in 2016-2017 (in Thai) 2017.
3. Jain R, Sontisirikit S, Iamsirithaworn S, Prendinger H. Prediction of dengue outbreaks based on disease surveillance, meteorological and socio-economic data. BMC Infect Dis. 2019;19(1):272.
4. Sánchez-González G, Condé R, Noguez Moreno R, López Vázquez PC. Prediction of dengue outbreaks in Mexico based on entomological, meteorological and demographic data. PLoS One. 2018;13(8):e0196047.
5. Piedrahita LD, Agudelo Salas IY, Marin K, Trujillo AI, Osorio JE, Arboleda-Sanchez SO, et al. Risk factors associated with dengue transmission and spatial distribution of high seroprevalence in schoolchildren from the urban area of Medellin, Colombia. Can J Infect Dis Med Microbiol. 2018;2018:2308095.
6. Sirisena P, Noordeen F, Kurukulasuriya H, Romesh TA, Fernando L. Effect of climatic factors and population density on the distribution of dengue in Sri Lanka: A GIS based evaluation for prediction of outbreaks. PLoS One. 2017;12(1):e0166806.
7. Schmidt WP, Suzuki M, Thiem VD, White RG, Tsuzuki A, Yoshida LM, et al. Population density, water supply, and the risk of dengue fever in Vietnam: cohort study and spatial analysis. PLoS medicine. 2011;8(8):e1001082.
8. Alvarado-Castro V, Paredes-Solís S, Nava-Aguilera E, Morales-Pérez A, Alarcón-Morales L, Balderas-Vargas NA, et al. Assessing the effects of interventions for Aedes aegypti control: Systematic review and meta-analysis of cluster randomised controlled trials. BMC public health. 2017;17(Suppl 1):384.
9. Suwanbamrung C, Thoutong C, Eksirinimit T, Tongjan S, Thongkew K. The use of the "Lansaka Model" as the larval indices surveillance system for a sustainable solution to the dengue problem in southern Thailand. PLoS One. 2018;13(8):e0201107-e.
10. Banerjee S, Aditya G, Saha GK. Household Wastes as Larval Habitats of Dengue Vectors: Comparison between Urban and Rural Areas of Kolkata, India. PLoS One. 2015;10(10):e0138082-e.
11. Bowman LR, Runge-Ranzinger S, McCall PJ. Assessing the Relationship between Vector Indices and Dengue Transmission: A Systematic Review of the Evidence. PLoS Neglected Tropical Diseases. 2014;8(5):e2848; 1-11.
12. Shen JC, Luo L, Li L, Jing QL, Ou CQ, Yang ZC, et al. The impacts of mosquito density and meteorological factors on dengue fever epidemics in Guangzhou, China, 2006–2014: A time-series analysis. Biomed Environ Sci. 2015;28(5):321-9.
13. Suwanbamrung C, Dumpan A, Thammapalo S, Sumrongtong R, Phedkeang P. A model of community capacity building for sustainable dengue problem solution in Southern Thailand. Health. 2011;3(9):584-601.
